# The Arrangements at Edinburgh and Dundee

**Published:** 1914-08-15

**Authors:** 


### Preparations in Scotland.

THE ARRANGEMENTS AT EDINBURGH AND DUNDEE.

### Edinburgh and District.

The newspapers record that Mr. Alfred Mosley has offered to build and equip what is described as a Base hospital for the reception of naval patients, to be situated at South Queensferry. It is to contain 64 beds only, and cannot therefore be a Base hospital in any event, for to be so it should contain at least 500 beds. It was announced on the 11th inst. that the directors of the Royal Infirmary, Edinburgh, had consented to make that great institution into a Base hospital during the war. We believe the facts to be that the Mosley Hospital has been presented to Her Majesty the Queen and Princess Christian, and accepted by them on behalf of the Admiralty. We understand it is to be worked in conjunction with the already established Naval Hospital at Rosyth, which may account for the use of the term "Base" hospital in this connection. The staff is left entirely in the hands of the donor, who, it is reported, is desirous of employing the same staff as he took with him to the South African war.

### Edinburgh Royal Infirmary as a Base Hospital.

With many other hospitals the Edinburgh Royal Infirmary is considerably crippled owing to the depletion in the medical staff, so many of their physicians and surgeons having been called up for service. Lieutenant-Colonel Sir Joseph Fayrer, Bart., medical superintendent of the infirmary, is in command of the 2nd Scottish General Hospital. There are also eighteen physicians and surgeons of the infirmary attached to this corps, all of whom are now on duty. Several other members of the staff are on Territorial service. There has been quite a rush of the younger doctors (residents, etc.) to volunteer for active medical service; but, as is easily understood, their services are urgently required at home, as it is quite possible the Royal Infirmary will become a Base hospital for the wounded.

### Dundee.

The members of the Dundee Branch of the British Red Cross Society are reported to be making preparations in case their services may be required. The managers of the Dundee Royal Infirmary have offered the Admiralty the use of 155 beds, which offer has been accepted, and the Corporation of Dundee has placed the Caird Home of Rest at the disposal of the Red Cross Society, which is to be utilised as a Temporary hospital. We regret to have to record, however, that at present the Dundee Branch is very much handicapped by the want of money to carry on its work. Hurry up, Dundee!

